# 2D Optical Gratings Based on Hexagonal Voids on Transparent Elastomeric Substrate

**DOI:** 10.3390/mi9070345

**Published:** 2018-07-10

**Authors:** Valentina Piccolo, Andrea Chiappini, Cristina Armellini, Mario Barozzi, Anna Lukowiak, Pier-John A. Sazio, Alessandro Vaccari, Maurizio Ferrari, Daniele Zonta

**Affiliations:** 1 DICAM-University of Trento, Via Mesiano 77, 38123 Trento, Italy; valentina.piccolo@unitn.it; 2 IFN-CNR CSMFO Lab & FBK CMM, Via alla Cascata 56/C, 38123 Trento, Italy; cristina.armellini@unitn.it; 3 CMM-MNF, Fondazione Bruno Kessler, Via Sommarive 18, 38123 Trento (Povo), Italy; barozzi@fbk.eu; 4 Institute of Low Temperature and Structure Research PAS, 50-422 Wroclaw, Poland; a.lukowiak@intibs.pl; 5 ORC, University of Southampton, University Road, Southampton SO17 1BJ, UK; P.A.Sazio@soton.ac.uk; 6 CMM-ARES, Fondazione Bruno Kessler, Via Sommarive 18, 38123 Trento (Povo), Italy; vaccari@fbk.eu; 7 Enrico Fermi Centre, Piazza del Viminale 1, 00184 Roma, Italy; maurizio.ferrari@ifn.cnr.it; 8 Department of Civil and Environmental Engineering, University of Strathclyde, Montrose Street, 75, Glasgow G1 1XJ, UK; daniele.zonta@strath.ac.uk

**Keywords:** micro/nano patterning, 2D colloidal crystal, soft colloidal lithography, strain microsensor, vectorial strain gauge

## Abstract

A chromatic vectorial strain sensor constituted by hexagonal voids on transparent elastomeric substrate has been successfully fabricated via soft colloidal lithography. Initially a highly ordered 1.6 microns polystyrene spheres monolayer colloidal crystal has been realized by wedge-shaped cell method and used as a suitable mold to replicate the periodic structure on a polydimethylsiloxane sheet. The replicated 2D array is characterized by high periodicity and regularity over a large area, as evidenced by morphological and optical properties obtained by means of SEM, absorption and reflectance spectroscopy. In particular, the optical features of the nanostructured elastomer have been investigated in respect to uniaxial deformation up to 10% of its initial length, demonstrating a linear, tunable and reversible response, with a sensitivity of 4.5 ± 0.1 nm/%. Finally, it has been demonstrated that the specific geometrical configuration allows determining simultaneously the vectorial strain-stress information in the x and y directions.

## 1. Introduction

Among the different fabrication techniques that allow obtaining micro/nanostructured surfaces, colloidal lithography is attracting big interest due to low cost, time efficiency, simplicity, and the possibility to pattern over a large surface area [1]. 

This bottom-up approach exploits the self-assembly of hard dielectric micro and nano spheres such as silica or polystyrene (PS) in order to fabricate two dimensional arrays. In recent literature, 2D colloidal crystals have been realized by self-assembly under electrophoresis deposition [2], Langmuir–Blodgett deposition [3], spin coating [4] and capillary forces [5]. Considering this last approach, Sun et al. [6] have demonstrated that the use of wedge-shaped cell allows obtaining large domains 2D colloidal crystals, with centimeter size, taking advantage on the capillary forces and drying front formed in the cell. 

In this contest it is worth mentioning that 2D colloidal crystals are interesting and promising systems for micro and nanopatterning due to their periodicity and specific size [7]. In micro and nano patterning field, colloidal crystals can be employed as lithographic masks or as molds for the production of micro and nanostructures for light trapping applications [8] or for the realization of SERS (Surface Enhanced Raman Spectroscopy) substrates [9]. Furthermore, they can act as masters by means of soft lithography in order to produce hexagonally arrayed structures. 

These types of systems can be employed for the realization of responsive materials able to measure physical quantities such as magnetic fields [10], and temperature [11], or detect different chemicals, [12,13,14] including important analytes such as glucose [15], creatinine [16] and nerve gas agents [17].

Focusing the attention on mechanical parameters (i.e., strain), periodic polymeric photonic materials demonstrated sensitivity to deformation, in particular different configurations have been employed such as opal-type photonic crystals infiltrated with elastomeric materials [18,19]; 1D grating based on buckled thin film with periodic sinusoidal patterns on a transparent elastomeric substrate [20]; 1D array of gold nanoparticles on flexible substrate [21] and double sided 1D orthogonal polydimethylsiloxane (PDMS) gratings [22].

In particular, the realization of surface stress-based sensors has become fundamental in several fields in order to detect acoustic waves and forces on different structures such as spacecrafts, submarines, buildings or bridges. 

Recently Guo et al. [22] have demonstrated that a double sided 1D orthogonal polydimethylsiloxane grating can be used as a vector mechanical sensor, able to detect mechanical parameters and giving information about their direction and strength. 

In this work we have developed a strain/stress vector sensor based on hexagonal voids on a transparent elastomeric substrate: due to the specific geometric configuration, the application of a horizontal strain induces an opposite movement of the diffraction spots created by a white light impinging on the structure. The relative displacement of these spots can be investigated to estimate the vectorial strain/stress information and to characterize the applied strain in both the x and y directions. This structure paves the way for the development of low cost vector strain sensor systems.

## 2. Materials and Methods

### 2.1. Materials

The PS latex beads were delivered by Thermo Scientific (Waltham, MA, USA), the PDMS Sylgard 184 by Dow Corning (Midland, MI, USA) and all the chemicals (Absolute ethanol, Clorothrimethylsilane and Dimethylformamide), used as received, by Aldrich (St. Louis, MO, USA).

### 2.2. PS Colloidal Particles and Substrate Preparation

Monodisperse latex particles 1.6 microns in diameter and size distribution of 0.021 µm, 1.3% CV were purchased from Thermo Scientific and used as received at standard concentration of 1 wt% suspension in water. The v-SiO_2_ substrates were cleaned firstly by brushing with neutral glassware detergent and then by ethanol. Finally, they were treated in an ozone cleaner for 30 min.

### 2.3. Assembly of the PS 2D Template

The PS spheres monolayer was used as a template for the fabrication of the PDMS grating and was deposited on v-SiO_2_ by means of the wedge-shaped cell method. This growth method allows the deposition of large domains two-dimensional colloidal crystals that self-organize by controlling the drying front of evaporation when the constituting particles are confined within two slides holding at an angle of about 2°. After the infiltration of 125 µL of PS suspension, the cell was maintained at room temperature (RT) and relative humidity (RH) of 40% for 1 day. Due to the evaporation of the solvent in the suspension, the latex beads crystallized in an ordered hexagonal structure.

### 2.4. Functionalization and Infiltration of the PS 2D Template

The 2D PDMS grating was obtained by infiltrating the template with the elastomer. Before the infiltration, to facilitate the following peeling off from the glassy substrate, the PS monolayer was functionalized by silanization with clorotrimethylsilane in a Petri dish for 90 min. As a second step a mixture of a 10:1 base:curing Sylgard 184 elastomer was poured on the functionalized template and thermally cured for 4 h at 65 °C. Finally, the PDMS with embedded PS spheres was gently removed from the glass substrate by peeling off the elastomer.

### 2.5. PS Particles Chemical Etching

The last step of the fabrication protocol was the etching of the PS particles in the elastomeric matrix that was performed by immersion of the PDMS slab in dimethylformamide for 90 min. Dimethylformamide is a solvent for PS and a non-solvent for PDMS, hence it provides a selective etching of the latex beads allowing the formation of an inverse replica of the template based on hexagonal voids in elastomeric matrix. After the etching process the sample was rinsed in water and blown with nitrogen.

### 2.6. Sample Characterization

**Figure 1:**
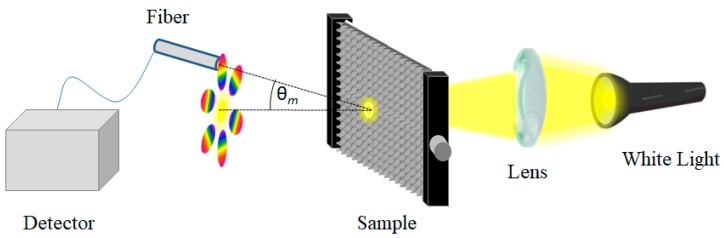
Sketch of the experimental set-up for the 2D diffraction grating measurements.

Morphological investigation of the samples has been carried out by means of scanning electron microscopy (SEM) measurements using a SEM JEOL JSM 7401-F FEG (Akishima, Tokyo, Japan). Transmittance measurements have been performed using a double beam VIS-NIR spectrophotometer Varian Cary 5000 (Palo Alto, CA, USA) in the range between 1000 and 2500 nm. The spectra of the samples were obtained illuminating the whole sample with a white light (halogen lamp) and collecting the diffracted light using a fiber-optic UV-Vis spectrometer Ocean Optics USB 4000 (Edinburgh, UK) as shown in Figure 1. Measurement of wavelength shift has been performed analyzing the displacement of two different diffraction spots, under the application of a horizontal strain. For both spots, this effect has been investigated by means of the wavelength shift of the 1st order of the transmittance diffraction, keeping the detection angle fixed at a specific value in order to have an initial spectrum centered in the visible region.

## 3. Results and Discussion

**Figure 2:**
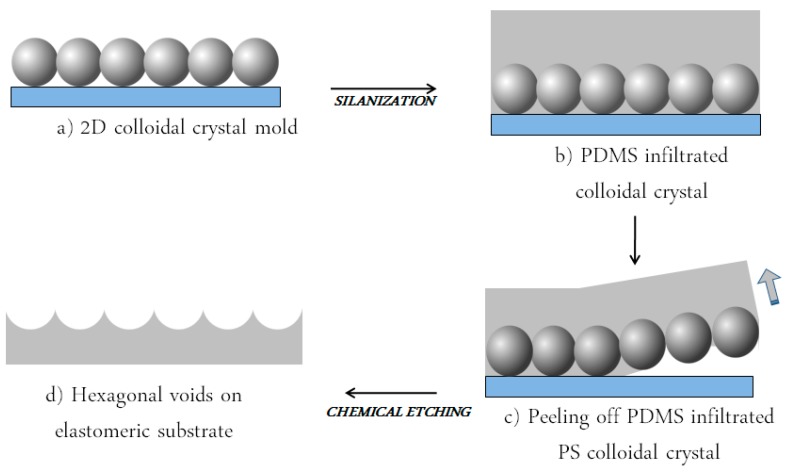
Schematic illustration of the experimental approach employed for the realization of 2D PDMS replica patterns (**a**) formation of 2D colloidal crystal by means of wedge-shaped cell (**b**) functionalization and infiltration of PDMS by capillary force; (**c**) peeling off PDMS infiltrated PS colloidal crystal; (**d**) chemical etching of the PS spheres.

**Figure 3:**
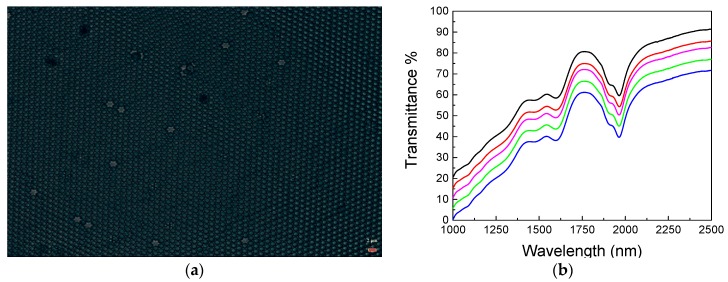
(**a**) Optical microscopy image of a typical area of the 2D colloidal crystals self-assembled using a wedge-shaped cell (scale bar of 2 μm). (**b**) Transmittance spectrum obtained on a 2D colloidal crystal deposited on a v-SiO_2_ substrate. The individual spectra are offset vertically by 5% for clarity (the black spectrum is the original one).

The first step, as shown in Figure 2a, concerned the realization of two-dimensional assembly of PS colloidal particles later used as a mold; Figure 3a reports a typical optical image of an ordered 2D colloidal crystal obtained by wedge-shaped cell method, where we can notice the presence of large areas of ordered domains (about 100 × 70 μm with few punctual defects). In Figure 3b, three transmission dips at λ = 1954 nm, 1598 nm, 1480 nm can be distinguished at normal incidence (*θ* = 0°) and are the result of the excitation of the photonic eigenmodes of the periodic dielectric structure due to its coupling with the incident light as proposed by Sun et al. [6], which, confirm the high optical quality. Furthermore, we can notice a decrease in the transmittance values attributed to an increase in the scattered radiation for lower wavelength affecting the collection of the zero order transmitted signal.

Moreover, the similar results acquired on different points, reported in Figure 3b, indicate the good homogeneity of the 2D colloidal crystal mold.

**Figure 4:**
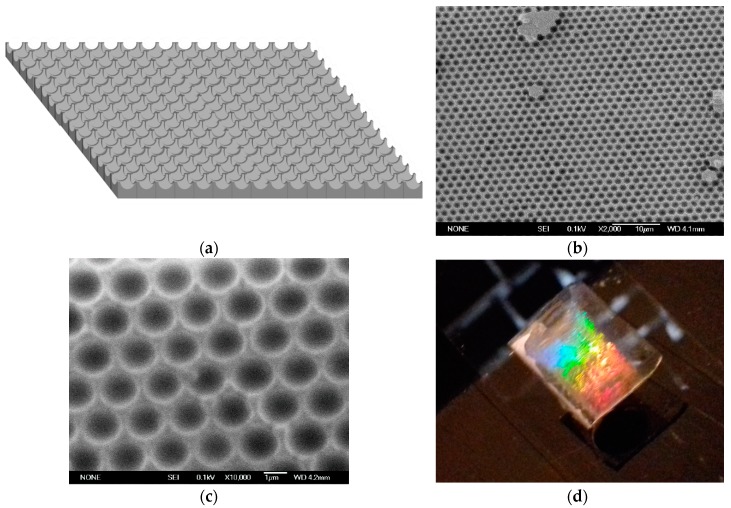
(**a**) Sketch of the concave structure obtained via soft lithography (not in scale) (**b**) SEM surface image of PDMS inverted colloidal crystal. (**c**) detail of the ordered hexagonal array. (**d**) Photograph of hexagonal voids on transparent elastomeric substrate.

Following the procedure described in Figure 2, a hexagonal voids regular structure (sketched in Figure 4a), has been fabricated via soft colloidal lithography. Figure 4b,c shows SEM-images of the resulting patterned polymeric structure where the reciprocal morphology of the PS mold has been successfully obtained. From a morphological point of view the hexagonal voids structure presents a periodicity of about 1600 nm with a depth of the voids of about 460 nm.

Furthermore, as shown in Figure 4d, the hexagonal voids structure, that can be seen as a 2D grating, presents an iridescent color that is attributed to the high order over a large area. In this case the morphology of the periodic hexagonal pattern satisfies the diffraction features that can be expressed through the simple law of diffraction (Equation (1)).

(1) $$n\cdot \sin\left(\theta_{m}\right)-n_{i}\cdot \sin\left(\theta_{i}\right)=\frac{m\cdot \lambda }{d}$$

where *θ_i_* is the incident angle while *θ_m_* corresponds to the *m*th diffraction order angle; *n_i_* and *n* are the refractive indices of the incident medium and of the medium where the diffracted orders propagate respectively; *λ* represents the wavelength of the incident light; and *d* is the period of the grating. 

**Figure 5:**
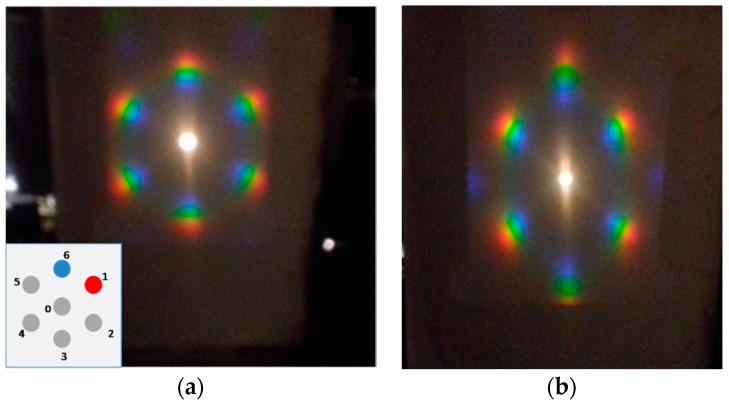
Strain induced diffraction spot movements: (**a**) Optical diffraction pattern without strain; inset: labelling of the investigated spots (1 and 6); (**b**) optical diffraction with a strain (ε) ε = 10% along the horizontal direction.

From an optical point of view illuminating the grating by white light, and collecting the diffraction projected on a screen, we can clearly notice the presence of a chromatic hexagonal pattern (see Figure 5) due to the arrangement of the semispherical voids.

In order to verify the optical response of the system to mechanical deformation, the structure has been mounted on a linear stage and a deformation in the horizontal direction was applied. 

As evidenced in Figure 5b, the application of a horizontal strain produces a change in the diffraction pattern. In this case it is worth mentioning that the movement of the first-order diffraction spot (see points 1 and 6 as labeled in the inset) is attributed to the variation of the grating period as a function of the strain, as predicted by the multi-slit Fraunhofer diffraction theory.

In particular comparing Figure 5a,b, focusing the attention on spot number 1, we can observe its movement towards the center (0), while if we consider spot number 6 we can notice that it moved away from the zero order. This effect can be attributed to an increase in the diffraction pitch in the parallel direction of the strain, and a consequent decrease (contraction) in the opposite side. 

These features have been investigated by means of reflectance measurements detecting the wavelength shift of the 1st order of the transmittance diffraction, maintaining fixed the detector and applying a different strain to the grating.

**Figure 6:**
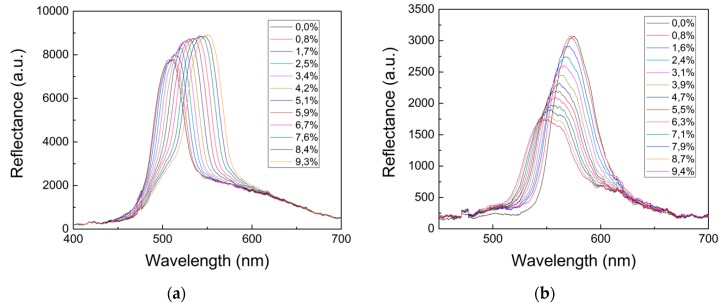
Reflectance spectra collected considering: (**a**) spot 1 as a function of the applied strain; (**b**) spot 6 as a function of the applied strain.

Analyzing Figure 6a, related to spot number 1, we can notice that the first order of the diffraction peak presents a noticeable red-shift when increasing the applied strain. The diffraction peak wavelength passes from 510 to 553 nm for a uniaxial deformation of the structure up to 10% of its initial length. On the other hand, for spot 6 we have observed a decrease in wavelength of the diffraction peak from 575 to 551 nm. The images shown in Figure 5 and the difference in the peak wavelength shifts indicate that the strain induces an elliptical modification of the voids. Evidently, Figure 5 is suggestive of the fact that the grating’s sensitivity is much higher against longitudinal geometrical changes then transversal ones. Indeed, the former are due to the imposed strain while the latter are due to Poisson’s effect. Clearly, the initially circular semi-voids become elongated ellipses in the direction of the applied strain. 

Moreover, we can see an increase of the intensity of the transmitted diffracted light at longer wavelength. To explain this effect, we have devised a simple model of its optical response. According to this model, the grating optical behavior is assimilable to two 2D arrays of secondary sources, both having the periodicity of the hexagonal semi-voids structure. The two arrays however, are half shifted in the grating plane, because one of them corresponds to rays emerging from the semi-void tops and the other corresponds to rays emerging from the semi-void bottoms. The two kinds of rays have an inherent optical path length difference due to the difference in the top and bottom substrate height. After calculations, the resulting intensity pattern for the downstream interference formula is thus depending from the primary beam wavelength, and in such a way that at an increase of its value necessarily implies an increase in the revealed intensity of a given secondary maximum [23].

**Figure 7:**
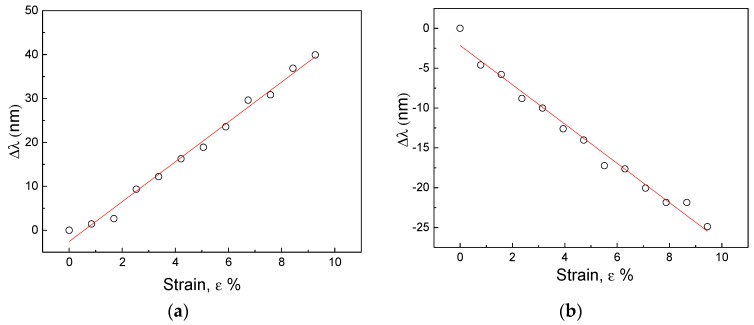
Experimental relationship between the peak position of the diffracted light (**a**) for spot 1 and (**b**) spot 6 in respect of the strain as a result of the elongation tests, (error bars are hidden by the circle points).

Now in order to determine the sensitivity of the 2D grating as strain sensor we have analyzed the variation in wavelength of the diffraction peak as a function of the applied strain. In Figure 7 we report the variation of the peak positions of the diffracted light (a) for spot 1 and (b) spot 6 in respect to the % applied strain. First of all, we can notice a linear behavior, moreover we can determine a sensitivity equal to 4.5 ± 0.1 nm/% and 2.5 ± 0.1 nm/% for spot 6 and 1 respectively. These results, if compared with those reported in the literature for mechanochromic systems, permit to include the developed structure among the most sensitive as strain sensors [21].

## 4. Conclusions

A chromatic strain sensor based on hexagonal voids on a transparent PDMS elastomeric substrate has been realized via soft colloidal lithography. The fabricated 2D grating can be employed for the development of a low cost and innovative sensor able to determine simultaneously the vectorial strain-stress information in the x and y directions. 

Moreover, we have demonstrated that the sensor exhibits a tunable and reversible response under the application of a mechanical strain. 

Optical reflection measurements have evidenced a linear behavior under the application of a horizontal strain up to 10% of its original length. The sensitivity of 4.5 ± 0.1 nm/%, when compared with mechanochromic photonic systems already present in literature, permits to classify the structure developed among the most sensitive strain sensors, paving the way for its applications in several fields such as smart sensing, mechanical sensing, and strain imaging.
